# Clinical outcome for heart failure hospitalizations in patients with leadless pacemaker

**DOI:** 10.1002/joa3.12761

**Published:** 2022-07-28

**Authors:** Tomonori Katsuki, Michio Nagashima, Hiroyuki Kono, Yohei Sadohara, Jun Hirokami, Rei Kuji, Kengo Korai, Masato Fukunaga, Kenichi Hiroshima, Kenji Ando

**Affiliations:** ^1^ Department of Cardiology Kokura Memorial Hospital Kitakyushu Japan

**Keywords:** heart failure hospitalization, leadless pacemaker

## Abstract

**Introduction:**

The long‐term performance of leadless pacemaker (LPM) has not been well evaluated.

**Methods:**

Between September 2017 and January 2021, 929 consecutive patients who underwent pacemaker implantation were grouped according to the types of pacemakers: LPM (LPM group, *n* = 368) and conventional pacemaker (PM group, *n* = 561).

**Results:**

The median follow‐up duration was 1.7 years (interquartile range 0.8–2.6 years). Hospitalization rate for heart failure in the LPM group was 9.3%, 15.6%, and 21.6% at 1, 2, 3 years, respectively. The LPM group had a significantly higher adjusted heart failure hospitalization risk than the PM group [hazard ratio (HR) 1.70, 95% confidence interval (CI) 1.09–2.64, *p* = .01]. More patients with symptomatic bradycardia caused by sinus node dysfunction (SND) in the LPM group (*n* = 150) were admitted to the hospital for heart failure compared to those in the PM group (*n* = 219) (HR 2.02, 95%CI 1.04–3.90, *p* = .03), whereas no significant difference was observed between the two groups in the patients with bradycardia caused by atrial fibrillation (LPM group, *n* = 71; PM group, *n* = 18) or atrioventricular block (LPM group, *n* = 147; PM group, *n* = 324).

**Conclusions:**

Patients who received LPM implantation had greater hospitalization risk for heart failure, compared to those who received conventional pacemaker implantation. The increased risk was mainly attributed to patients with SND.

## 1 INTRODUCTION

Leadless pacemakers (LPMs) have been associated with lower risk of complications such as infections, pneumothorax, and skin erosion,^1^, ^2^, ^3^ and they have been considered as a good option for elderly patients.^4^ Ventricular pacing has been shown to induce ventricular desynchronization, which can cause heart failure.^5^, ^6^, ^7^ A recent study reported LPM therapy was associated with increased tricuspid valve dysfunction.^8^ Although the results suggested an increased risk of heart failure, the incidence of heart failure in patients with LPM has not been well evaluated. Therefore, the present study aimed to investigate the association between hospitalization due to heart failure and LPM implantation.

## 2 METHODS

### 2.1 Study population

This single‐center, retrospective, and observational study was conducted between September 2017 and January 2021, and it included 929 consecutive patients who underwent pacemaker implantation at Kokura Memorial Hospital, Kitakyushu, Japan. Among the identified patients, 368 underwent LPM implantation (Micra VR™, Medtronic, Inc, Minneapolis, Minnesota). In accordance with the Japanese guideline focused update,^9^ LPM implantation was performed when venous access should be preserved or when venous occlusion or stenosis was identified. Furthermore, for patients with sinus node dysfunction (SND) and atrioventricular block (AVB), LPM implantation was considered when it was thought to be more beneficial outperforming the limitations of VVI mode, because of severe frailty and less than 1‐year survival rate, and when the LPM would be a better pacing option in patients with history of cardiac implantable electronic device infection.^3^ All patients provided informed consent before the initiation of the procedure. Moreover, they were provided the opportunity to choose between the LPM and the conventional transvenous pacemaker (PM) implantation.

The study protocol was approved by the institutional review board of Kokura Memorial Hospital, and the study was conducted in accordance with the Declaration of Helsinki. Obtaining written informed consent from the patients was waived because of the retrospective nature of the study, but we excluded those who refused participation when contacted for follow‐up. This strategy was in concordance with the guidelines of the Japanese Ministry of Health, Labor and Welfare.

### 2.2 Study outcome and follow up

The clinical outcome was hospitalization due to heart failure. Hospitalization was considered when patients presented with signs and symptoms consistent with heart failure and required intravenous therapy, whereas the indication for hospitalization due to heart failure was left to the physician's discretion. Follow‐up visits were planned at 1, 3, 12, 24, and 36 months after pacemaker implantation. Clinical follow‐up data were obtained from medical records and/or via telephone contact with the patients, families, or referring physicians.

### 2.3 Statistical analysis

Continuous variables were presented as means ± standard deviations and compared among groups using Student's *t*‐test, whereas categorical variables were compared using Fisher's exact test. Moreover, the sub‐analysis was also performed based on the background disease for which pacing indications, such as SND, AVB, and bradycardic atrial fibrillation (AF). Cumulative incidences of clinical outcomes were estimated using Kaplan–Meier curves. Crude and adjusted hazard ratios (HRs) with 95% confidence intervals (CI) were calculated using univariate and multivariable Cox regression models. The following clinically relevant explanatory variables were included in the multivariable models to adjust for baseline characteristics: age, sex, body mass index, diabetes, hypertension, dyslipidemia, history of hospitalization for heart failure, left ventricular ejection fraction. All analyses were performed using the JMP statistical software (version 14.2.0; SAS Institute, Cary, NC, USA), with two‐sided *p* < .05 indicating statistical significance.

## 3 RESULTS

### 3.1 Baseline characteristics

Patient characteristics are summarized in Table 1. The mean age of the study population was 80.1 ± 9.4 years (range: 31–101 years) and 49% were male. Majority of patients had preserved left ventricular ejection fraction (61.1% ± 9.8%), and 67% of the patients were categorized as class I, based on New York Heart Association (NYHA) functional classification. Meanwhile, one‐fifth of patients had a history of hospitalization for heart failure. Pacing indications included SND (40%), AVB (50%), and bradycardic AF (10%).

**TABLE 1:** Patient characteristics

	Overall (*n* = 929)	LPM group (*n* = 368)	PM group (*n* = 561)	*p* value
Age, years	80.1 ± 9.4	84.7 ± 7.1	77.0 ± 9.5	<.0001
Male	454 (49%)	176 (48%)	278 (50%)	.61
Body mass index, kg/m^2^	22.6 ± 3.5	21.8 ± 3.6	23.2 ± 3.4	<.0001
NYHA functional classification				
I	627 (67%)	254 (69%)	373 (66%)	.42
II	224 (24%)	83 (23%)	141 (25%)	.37
III	68 (7%)	27 (7%)	41 (7%)	.99
IV	10 (1%)	4 (1%)	6 (1%)	.98
Diabetes	139 (15%)	53 (14%)	86 (15%)	.70
Hypertension	616 (66%)	241 (65%)	375 (67%)	.67
Dyslipidemia	303 (33%)	103 (28%)	200 (36%)	.01
Coronary artery disease	156 (17%)	53 (14%)	103 (18%)	.11
Cardiomyopathy	42 (5%)	14 (4%)	28 (5%)	.39
History of hospitalization for heart failure	188 (20%)	84 (23%)	104 (19%)	.11
Left ventricular ejection fraction, %	61.1 ± 9.8	60.0 ± 11.2	61.9 ± 8.7	.99
Pacing indications				
Sinus node dysfunction	369 (40%)	150 (41%)	219 (39%)	.60
Atrioventricular block	471 (50%)	147 (40%)	324 (58%)	<.0001
Atrial fibrillation	89 (10%)	71 (19%)	18 (3%)	<.0001
Medication				
Diuretic	247 (27%)	128 (35%)	119 (21%)	<.0001
ACE inhibitor	97 (10%)	49 (13%)	48 (9%)	.02
ARB	281 (30%)	100 (27%)	181 (32%)	.10
Mineralocorticoid receptor antagonist	138 (15%)	63 (17%)	75 (13%)	.12
β‐blocker	205 (22%)	96 (26%)	109 (19%)	.01
Calcium channel blocker	389 (42%)	141 (38%)	248 (44%)	.08
Antiarrhythmic drugs	32 (3%)	12 (3%)	20 (4%)	.80

Abbreviations: ACE, angiotensin converting enzyme; ARB, angiotensin II receptor blocker; NYHA, New York Heart Association.

Patients were divided into two groups according to the types of implanted pacemakers: the LPM group, which included 368 patients (40%), and the PM group, which included 561 patients (60%). Compared to the PM group, the LPM group were older and had lesser incidence rates of AVB but had higher incidence rates of bradycardic AF. Additionally, more patients in the LPM group were administered heart failure medication (diuretics, angiotensin converting enzyme, and β‐blockers) compared to those in the PM group.

To evaluate the interaction of age, we divided the patients into two groups according to median age (median 82 years, interquartile range 75–87 years: ≥82 years, *n* = 468; <82 years, *n* = 461). A total of 265 patients older than 82 years underwent LPM implantation, while a total of 103 patients younger than 82 years underwent LPM implantation (Tables S1 and S2).

Patient characteristics according to background disease are detailed in Tables S3–S5. In each subgroup, patients in the LPM group were relative older compared to those in the PM group. More AVB patients in the LPM group received diuretics and β‐blockers than those in the PM group. Moreover, half of the bradycardic AF patients in the PM group were categorized as New York Heart Association class III, whereas 72% had history of hospitalization for heart failure.

### 3.2 Clinical outcomes

**FIGURE 1:**
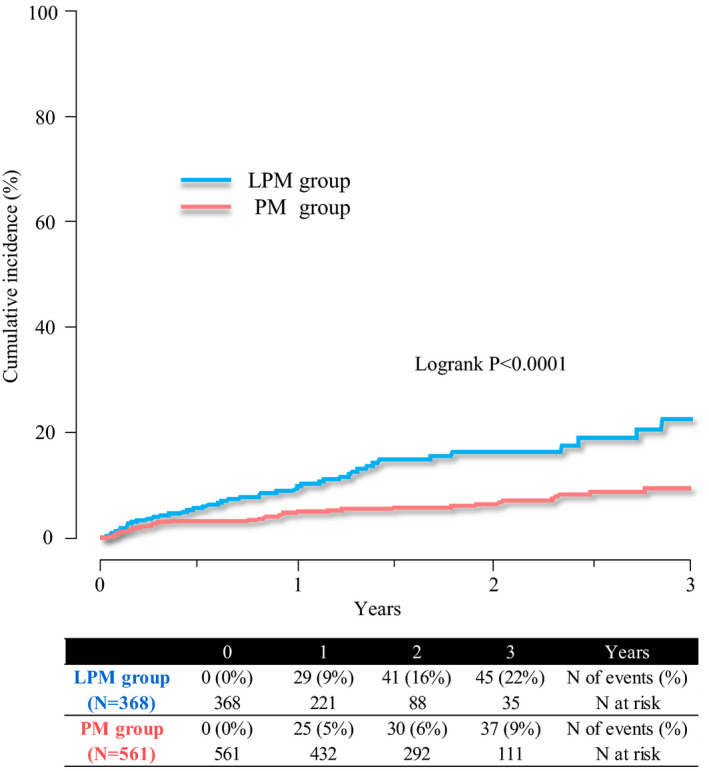
Kaplan–Meier curves for heart failure hospitalization.

**FIGURE 2:**
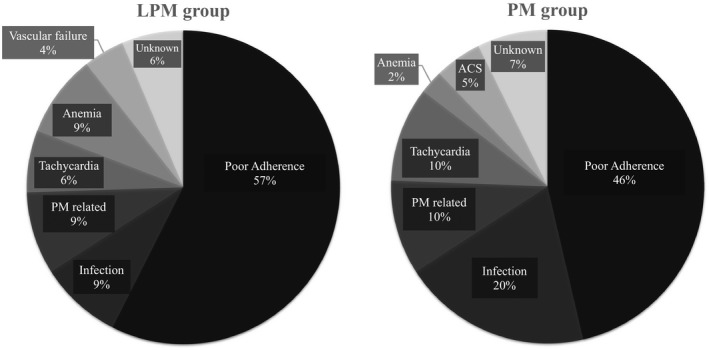
Causes of worsening heart failure.

The median follow‐up duration was 1.7 years (interquartile range 0.8–2.6 years). During the follow‐up period, 47 and 41 patients in the LPM and PM groups were hospitalized for heart failure, respectively. Hospitalization rates for heart failure was significantly higher in the LPM group than in the PM group (log‐rank *p* < .0001; Figure 1), with the difference between both groups being significant both before (crude HR 2.37, 95%CI 1.55–3.61, *p* < .001) and after adjusting for baseline characteristics (adjusted HR 1.69, 95%CI 1.08–2.62, *p* = .02, Table 2). A total of 88 patients were hospitalized for heart failure during the follow‐up period. The causes of worsening heart failure are summarized in Figure 2. Poor adherence and infection were the main causes of worsening heart failure, accounting for 66% in each group. Notably, desynchronization due to non‐physiological pacing or worsening tricuspid regurgitation due to the pacemaker leads was considered as a possible cause for worsening heart failure in 9% and 10% of the patients in the LPM and PM groups, respectively.

**TABLE 2:** Univariate and multivariable cox Hazard models for heart failure hospitalization

	Univariate		Multivariable	
	HR (95% CI)	*p* value	HR (95% CI)	*p* value
LPM group	2.37 (1.55–3.61)	<.001	1.69 (1.08–2.62)	.02
Age (80 ≥ years old)	2.42 (1.51–3.88)	<.001	2.60 (1.56–4.33)	<.001
Male	0.85 (0.56–1.30)	.46	1.01 (0.65–1.56)	.96
Body mass index (22 ≥ kg/m^2^)	0.79 (0.52–1.21)	.28	0.92 (0.60–1.42)	.72
Diabetes	1.66 (1.01–2.71)	.04	1.70 (1.02–2.84)	.04
Hypertension	0.95 (0.61–1.47)	.81	0.69 (0.44–1.09)	.11
Dyslipidemia	1.30 (0.84–2.01)	.22	1.49 (0.95–2.33)	.07
History of hospitalization for heart failure	3.63 (2.38–5.52)	<.001	3.14 (2.01–4.91)	<.001
Left ventricular ejection fraction (50 < %)	3.95 (2.42–6.46)	<.001	2.79 (1.66–4.70)	<.001

Abbreviation: LPM, leadless pacemaker.

A significant interaction was observed between the two groups established according to median age (*p* for the interaction .002). Among the ≥82 years patients, significantly more patients in the LPM group were hospitalized for heart failure than those in the PM group (adjusted HR 2.13, 95%CI 1.21–3.77, *p* = .008), whereas there was no similar significant difference in the <82 years patients (adjusted HR 1.36, 95%CI 0.61–3.02, *p* = .44).

Among patients with SND, the crude and adjusted risks of hospitalization for heart failure were significantly greater in the LPM group than in the PM group (crude HR 2.50, 95%CI 1.37–4.57, *p* = .002; adjusted HR 2.01, 95%CI 1.04–3.90, *p* = .03, Figure S1A and Table S6). Meanwhile, no significant differences were observed between the two groups among AVB patients (crude HR 1.68, 95%CI 0.79–3.56, *p* = .17; adjusted HR 1.30, 95%CI 0.58–2.91, *p* = .51, Figure S1B and Table S7), and among bradycardic AF patients (crude HR 1.51, 95%CI 0.42–5.42, *p* = .52; adjusted HR 2.14, 95%CI 0.43–10.65, *p* = .35, Figure S1C and Table S8). As shown in Figure S2A–C, poor adherence or infection in each subgroup caused majority of the patients to be hospitalized for heart failure. Among patients with SND indicated for pacing, the pacemaker‐related causes such as desynchronization due to non‐physiological pacing or worsening tricuspid regurgitation due to the pacemaker leads were observed in 13% and 10% of patients in the LPM and PM groups, respectively, whereas among patients with AVB for which pacing was indicated, the foretokened events were observed in 9% and 11% of patients in the LPM and PM groups, respectively. Obvious pacemaker‐related causes were not observed in patients with bradycardic AF for which pacing was indicated. Tachycardia, including atrial tachycardia and fibrillation, occurred in seven patients who were hospitalized for heart failure; of these, six were those with SND and one was an AF patient with bradycardia.

## 4 DISCUSSION

The salient findings of the present study were as follows: Hospitalization risk of heart failure was 1.69 times higher in patients who underwent LPM implantation than in those who underwent PM implantation. The main causes of worsening heart failure were poor adherence and infection, whereas approximately 10% of the causes were pacemaker‐related, irrespective of the types of pacemakers. Regarding the clinical endpoint, older patients (≥82 years) showed a trend similar to that in the overall population, though significant differences were not observed in younger patients (<82 years). According to disease background, hospitalization rates for heart failure were significantly higher in patients who underwent LPM implantation owing to SND compared to those who underwent PM implantation. Notably, no differences between the two devices were observed among patients who underwent pacemaker implantation owing to AVB and bradycardic AF. Tachycardia caused by worsening heart failure mainly occurred in SND patients.

Ventricular desynchronization caused by ventricular pacing increases the risk of hospitalization for heart failure. In particular, more than 40% cumulative ventricular pacing was a strong predictor of hospitalization due to heart failure in SND patients.^6^, ^7^ In the current study, clinical outcomes were mainly driven by SND. Among SND patients, the risk of hospitalization for heart failure was significantly higher in those with more than 40% cumulative ventricular pacing than in those with less than 40% cumulative ventricular pacing (Figure S3). As shown in Table S9, the LPM group had significantly more SND patients with more than 40% cumulative ventricular pacing after pacemaker implantation compared to the PM group, up to 2 years. Generally, SND patients need only atrial pacing. PMs fulfill the demand by atrial pacing, whereas LPMs fulfill the demand by ventricular pacing. The differences in ventricular pacing rates and in the responses between the two devices could affect the clinical outcomes. SND patients after pacemaker implantation often suffered from AF, with the evidence showing an increased risk of AF after ventricular pacing.^6^, ^7^ In the current study, supraventricular tachycardia accounted for 14% of the cases of worsening heart failure among SND patients. In the patients with PMs, AF is easily detected by checking the pacemaker, and the immediate treatment, such as anti‐tachycardia pacing, catheter ablation, and medication is possible. While the detection of supraventricular arrythmia would be delayed in the patients with LPMs. The administration of antiarrhythmic drugs, including β‐blockers, is needed for AF management, whereas the administration of the drugs for SND patients with LPMs could worsen bradycardia and increase desynchronization due to non‐physiological ventricular pacing. Based on these findings, dual‐chamber pacemakers with atrial anti‐tachycardia pacing function^10^ would be a better option than LPMs for SND patients.

In the current study, LPMs were implanted for relative elderly and low body mass index patients; however, such patients often have a poor general condition and prognoses.^11^, ^12^ Our analysis on the interaction of age showed that those ≥82 years were more likely to have undergone LPM implantation, with such patients also having significantly higher hospitalization rates due to heart failure. The quantitative interaction of age could be induced by factors that are not listed in the baseline characteristics, such as frailty, dementia, and other comorbidities. Thus, elderly patients who underwent LPM implantation would need more careful treatment to prevent the occurrence of heart failure. Given the increasing risk of hospitalization due to heart failure among the elderly, leadless dual‐chamber pacing devices^13^ could be a better option for patients with a high risk for complications.^1^, ^2^, ^3^

### 4.1 Limitations

The current study has several limitations. First, this was a single‐center and retrospective study. Second, the clinical outcome was evaluated up to 4 years, and the follow‐up was far from complete. Third, we presented cumulative ventricular pacing data at the discussion section; however, all patients' assessment data could not be collected. Fourth, we corrected the difference between the two groups by performing the multivariable analysis and confirming the interaction based on the median value of age. However, due to the difference in the indication between the LPM and PM implantations, the baseline characteristics were completely different between the two groups.

## 5 CONCLUSIONS

Hospitalization risk for heart failure was higher in patients who underwent LPM implantation than in those who underwent PM implantation. The increased risk was mainly attributed to patients ≥82 years old, and those with SND for which pacing indication.

## FUNDING INFORMATION

None.

## CONFLICT OF INTEREST

Authors declare no conflict of interests for this article.

## ETHICS APPROVAL STATEMENT

The study protocol was approved by the institutional review board of Kokura Memorial Hospital, and the study was conducted in accordance with the Declaration of Helsinki.

## PATIENT CONSENT STATEMENT

We obtained informed written or verbal consent from all participants.

## CLINICAL TRIAL REGISTRATION

This study was approved by the institutional review board of Kokura Memorial Hospital. (Permission number: 21101301).

## Supporting information


Appendix S1
