# Supplementary material for: Clinical outcome for heart failure hospitalizations in patients with leadless pacemaker
Source: J Arrhythm. 2022 Jul 28;38(5):730–5. doi: 10.1002/joa3.12761 (PMC9535791; doi:10.1002/joa3.12761)
Supplement: Supplementary file 1 — Appendix S1 [file JOA3-38-730-s001.docx]

**SUPPLEMENTARY MATERIAL**

**Supplementary Table 1.**

|  | Overall | LPM group | PM group | P value |
| --- | --- | --- | --- | --- |
|  | (n = 468) | (n = 265) | (n =203) |  |
| Age, years | 87.2±3.9 | 88.1±4.0 | 86.0±3.4 | <0.0001 |
| Male | 209 (45%) | 114 (43%) | 95 (47%) | 0.41 |
| Body mass index, kg/m^2^ | 22.0±3.4 | 21.5±3.6 | 22.7±3.0 | <0.0001 |
| NYHA functional classification |  |  |  |  |
| - I | 294 (63%) | 177 (67%) | 117 (58%) | 0.04 |
| - II | 125 (27%) | 61 (23%) | 64 (32%) | 0.03 |
| - III | 41 (9%) | 23 (9%) | 18 (9%) | 0.94 |
| - IV | 8 (2%) | 4 (2%) | 4 (2%) | 0.70 |
| Diabetes | 61 (13%) | 31 (12%) | 30 (15%) | 0.32 |
| Hypertension | 328 (70%) | 175 (66%) | 153 (75%) | 0.02 |
| Dyslipidemia | 141 (30%) | 69 (26%) | 72 (35%) | 0.02 |
| Coronary artery disease | 82 (18%) | 36 (14%) | 46 (23%) | 0.01 |
| Cardiomyopathy | 18 (4%) | 10 (4%) | 8 (4%) | 0.92 |
| History of hospitalization for heart failure | 103 (22%) | 62 (23%) | 41 (20%) | 0.40 |
| Left ventricular ejection fraction, % | 60.6±11.2 | 59.6±12.4 | 61.9±9.1 | 0.02 |
| Pacing indications |  |  |  |  |
| - Sinus node dysfunction | 155 (33%) | 93 (35%) | 62 (31%) | 0.29 |
| - Atrioventricular block | 258 (55%) | 122 (46%) | 136 (67%) | <0.0001 |
| - Atrial fibrillation | 55 (12%) | 50 (19%) | 5 (2%) | <0.0001 |
| Medication |  |  |  |  |
| - Diuretic | 156 (33%) | 98 (37%) | 58 (29%) | 0.054 |
| - ACE inhibitor | 55 (12%) | 38 (14%) | 17 (8%) | 0.04 |
| - ARB | 153 (33%) | 76 (29%) | 77 (38%) | 0.03 |
| - Mineralocorticoid receptor antagonist | 85 (18%) | 52 (20%) | 33 (16%) | 0.34 |
| - β-blocker | 105 (22%) | 72 (27%) | 33 (16%) | 0.004 |
| - Calcium channel blocker | 212 (45%) | 107 (40%) | 105 (52%) | 0.01 |
| - Antiarrhythmic drugs | 19 (4%) | 9 (3%) | 10 (5%) | 0.40 |

**Supplementary Table 2.**

|  | Overall | LPM group | PM group | P value |
| --- | --- | --- | --- | --- |
|  | (n = 461) | (n = 103) | (n = 358) |  |
| Age, years | 72.8±7.7 | 75.8±5.5 | 72.0±8.1 | <0.0001 |
| Male | 245 (53%) | 62 (60%) | 183 (51%) | 0.10 |
| Body mass index, kg/m^2^ | 23.3±3.5 | 22.8±3.1 | 23.4±3.5 | 0.10 |
| NYHA functional classification |  |  |  |  |
| - I | 333 (72%) | 77 (75%) | 256 (72%) | 0.51 |
| - II | 99 (21%) | 22 (21%) | 77 (22%) | 0.97 |
| - III | 27 (6%) | 4 (4%) | 23 (6%) | 0.33 |
| - IV | 2 (%) | (%) | 2 (1%) | 0.44 |
| Diabetes | 78 (17%) | 22 (21%) | 56 (16%) | 0.17 |
| Hypertension | 288 (62%) | 66 (64%) | 222 (62%) | 0.70 |
| Dyslipidemia | 162 (35%) | 34 (33%) | 128 (36%) | 0.60 |
| Coronary artery disease | 74 (16%) | 17 (17%) | 57 (16%) | 0.88 |
| Cardiomyopathy | 24 (5%) | 4 (4%) | 20 (6%) | 0.49 |
| History of hospitalization for heart failure | 85 (18%) | 22 (21%) | 63 (18%) | 0.38 |
| Left ventricular ejection fraction, % | 61.7±8.2 | 61.1±6.9 | 61.8±8.5 | 0.45 |
| Pacing indications |  |  |  |  |
| - Sinus node dysfunction | 214 (46%) | 57 (55%) | 157 (44%) | 0.03 |
| - Atrioventricular block | 213 (46%) | 25 (24%) | 188 (53%) | <0.0001 |
| - Atrial fibrillation | 34 (7%) | 21 (20%) | 13 (4%) | <0.0001 |
| Medication |  |  |  |  |
| - Diuretic | 91 (20%) | 30 (29%) | 61 (17%) | 0.008 |
| - ACE inhibitor | 42 (9%) | 11 (11%) | 31 (9%) | 0.53 |
| - ARB | 128 (28%) | 24 (23%) | 104 (29%) | 0.25 |
| - Mineralocorticoid receptor antagonist | 53 (12%) | 11 (11%) | 42 (12%) | 0.76 |
| - β-blocker | 100 (22%) | 24 (23%) | 76 (21%) | 0.65 |
| - Calcium channel blocker | 177 (38%) | 34 (33%) | 143 (40%) | 0.20 |
| - Antiarrhythmic drugs | 13 (3%) | 1 (3%) | 10 (3%) | 0.94 |

**Supplementary Table 3.**

|  | Overall | LPM group | PM group | P value |
| --- | --- | --- | --- | --- |
|  | (n = 369) | (n = 150) | (n = 219) |  |
| Age, years | 79.4±8.3 | 79.4±8.3 | 76.6±8.0 | <0.0001 |
| Male | 152 (41%) | 55 (37%) | 97 (44%) | 0.14 |
| Body mass index, kg/m^2^ | 22.7±3.6 | 22.7±3.6 | 23.4±3.4 | <0.0001 |
| NYHA functional classification |  |  |  |  |
| - I | 276 (75%) | 118 (79%) | 158 (72%) | 0.16 |
| - II | 72 (20%) | 24 (16%) | 48 (22%) | 0.16 |
| - III | 19 (5%) | 8 (5%) | 11 (5%) | 0.89 |
| - IV | 2 (1%) | 0 (0%) | 2 (1%) | 0.24 |
| Diabetes | 46 (12%) | 16 (11%) | 30 (14%) | 0.39 |
| Hypertension | 251 (68%) | 105 (70%) | 146 (67%) | 0.50 |
| Dyslipidemia | 132 (36%) | 52 (35%) | 80 (37%) | 0.71 |
| Coronary artery disease | 48 (13%) | 20 (13%) | 28 (13%) | 0.88 |
| Cardiomyopathy | 17 (5%) | 5 (3%) | 12 (5%) | 0.33 |
| History of hospitalization for heart failure | 70 (19%) | 25 (17%) | 45 (21%) | 0.35 |
| Left ventricular ejection fraction, % | 61.1±9.1 | 61.1±9.1 | 62.3±6.5 | 0.99 |
| Medication |  |  |  |  |
| - Diuretic | 89 (24%) | 41 (27%) | 48 (22%) | 0.23 |
| - ACE inhibitor | 33 (9%) | 16 (11%) | 17 (8%) | 0.34 |
| - ARB | 119 (32%) | 48 (32%) | 71 (32%) | 0.93 |
| - Mineralocorticoid receptor antagonist | 46 (12%) | 16 (11%) | 30 (14%) | 0.39 |
| - β-blocker | 107 (29%) | 47 (31%) | 60 (27%) | 0.41 |
| - Calcium channel blocker | 165 (45%) | 65 (43%) | 100 (46%) | 0.65 |
| - Antiarrhythmic drugs | 19 (5%) | 6 (4%) | 13 (6%) | 0.41 |

**Supplementary Table 4.**

|  | Overall | LPM group | PM group | P value |
| --- | --- | --- | --- | --- |
|  | (n = 471) | (n = 147) | (n = 324) |  |
| Age, years | 80.2±10.5 | 86.0±7.6 | 77.5±10.6 | <0.0001 |
| Male | 244 (52%) | 76 (52%) | 168 (52%) | 0.97 |
| Body mass index, kg/m^2^ | 22.6±3.4 | 21.6±3.3 | 23.1±3.3 | <0.0001 |
| NYHA functional classification |  |  |  |  |
| - I | 310 (66%) | 100 (68%) | 210 (65%) | 0.50 |
| - II | 123 (26%) | 33 (22%) | 90 (28%) | 0.22 |
| - III | 33 (7%) | 12 (8%) | 21 (6%) | 0.51 |
| - IV | 5 (1%) | 2 (1%) | 3 (1%) | 0.67 |
| Diabetes | 78 (17%) | 23 (16%) | 55 (17%) | 0.72 |
| Hypertension | 313 (66%) | 96 (65%) | 217 (67%) | 0.72 |
| Dyslipidemia | 147 (31%) | 33 (22%) | 114 (35%) | 0.005 |
| Coronary artery disease | 93 (20%) | 22 (15%) | 71 (22%) | 0.08 |
| Cardiomyopathy | 22 (5%) | 7 (5%) | 15 (5%) | 0.95 |
| History of hospitalization for heart failure | 76 (16%) | 30 (20%) | 46 (14%) | 0.09 |
| Left ventricular ejection fraction, % | 61.1±10.7 | 60.0±12.4 | 61.6±9.9 | 0.92 |
| Medication |  |  |  |  |
| - Diuretic | 110 (23%) | 50 (34%) | 60 (19%) | 0.0002 |
| - ACE inhibitor | 49 (10%) | 20 (14%) | 29 (9%) | 0.13 |
| - ARB | 144 (31%) | 40 (27%) | 104 (32%) | 0.29 |
| - Mineralocorticoid receptor antagonist | 61 (13%) | 23 (16%) | 38 (12%) | 0.24 |
| - β-blocker | 81 (17%) | 35 (24%) | 46 (14%) | 0.01 |
| - Calcium channel blocker | 196 (42%) | 54 (37%) | 142 (44%) | 0.15 |
| - Antiarrhythmic drugs | 9 (2%) | 2 (1%) | 7 (2%) | 0.56 |

**Supplementary Table 5.**

|  | Overall | LPM group | PM group | P value |
| --- | --- | --- | --- | --- |
|  | (n = 89) | (n = 71) | (n = 18) |  |
| Age, years | 82.2±7.3 | 84.0±6.3 | 74.9±6 | <0.001 |
| Male | 58 (65%) | 45 (63%) | 13 (72%) | 0.48 |
| Body mass index, kg/m^2^ | 22.7±3.7 | 22.7±3.7 | 22.6±3.4 | 0.46 |
| NYHA functional classification |  |  |  |  |
| - I | 41 (46%) | 36 (51%) | 5 (28%) | 0.08 |
| - II | 29 (33%) | 26 (37%) | 3 (17%) | 0.11 |
| - III | 16 (18%) | 7 (10%) | 9 (50%) | <0.0001 |
| - IV | 3 (3%) | 2 (3%) | 1 (6%) | 0.57 |
| Diabetes | 15 (17%) | 14 (20%) | 1 (6%) | 0.15 |
| Hypertension | 52 (58%) | 40 (56%) | 12 (67%) | 0.43 |
| Dyslipidemia | 24 (27%) | 18 (25%) | 6 (33%) | 0.50 |
| Coronary artery disease | 15 (17%) | 11 (15%) | 4 (22%) | 0.50 |
| Cardiomyopathy | 3 (3%) | 2 (3%) | 1 (6%) | 0.57 |
| History of hospitalization for heart failure | 42 (47%) | 29 (41%) | 13 (72%) | 0.01 |
| Left ventricular ejection fraction, % | 61.2±7.4 | 61.5±6.7 | 60.3±9.9 | 0.33 |
| Medication |  |  |  |  |
| - Diuretic | 48 (54%) | 37 (52%) | 11 (61%) | 0.49 |
| - ACE inhibitor | 15 (17%) | 13 (18%) | 2 (11%) | 0.46 |
| - ARB | 18 (20%) | 12 (17%) | 6 (33%) | 0.12 |
| - Mineralocorticoid receptor antagonist | 31 (35%) | 24 (34%) | 7 (39%) | 0.69 |
| - β-blocker | 17 (19%) | 14 (20%) | 3 (17%) | 0.77 |
| - Calcium channel blocker | 28 (31%) | 22 (31%) | 6 (33%) | 0.85 |
| - Antiarrhythmic drugs | 4 (4%) | 4 (6%) | 0 (0%) | 0.30 |

**Supplementary Table 6.**

|  | Univariate | | Multivariable | |
| --- | --- | --- | --- | --- |
|  | HR (95% CI) | P value | HR (95% CI) | P value |
| LPM group | 2.50 (1.37-4.57) | 0.002 | 2.01 (1.04-3.90) | 0.03 |
| Age (80≥ years old) | 3.21 (1.65-6.25) | <0.001 | 3.10 (1.50-6.42) | 0.002 |
| Male | 0.82 (0.44-1.53) | 0.54 | 1.11 (0.58-2.12) | 0.74 |
| Body mass index (22≥ kg/m2) | 0.88 (0.48-1.60) | 0.68 | 0.90 (0.47-1.70) | 0.75 |
| Diabetes | 1.60 (0.74-3.45) | 0.22 | 1.62 (0.70-3.71) | 0.25 |
| Hypertension | 1.11 (0.58-2.12) | 0.74 | 0.75 (0.38-1.48) | 0.41 |
| Dyslipidemia | 1.24 (0.67-2.28) | 0.48 | 1.50 (0.78-2.86) | 0.22 |
| History of hospitalization for heart failure | 4.29 (2.37-7.76) | <0.001 | 4.22 (2.25-7.90) | <0.001 |
| Left ventricular ejection fraction (50< %) | 3.68 (1.81-7.46) | <0.001 | 2.36 (1.11-5.03) | 0.02 |

**Supplementary Table 7.**

|  | Univariate | | Multivariable | |
| --- | --- | --- | --- | --- |
|  | HR (95% CI) | P value | HR (95% CI) | P value |
| LPM group | 1.68 (0.79-3.56) | 0.17 | 1.30 (0.58-2.91) | 0.51 |
| Age (80≥ years old) | 2.00 (0.88-4.54) | 0.09 | 2.11 (0.86-5.14) | 0.09 |
| Male | 0.91 (0.44-1.89) | 0.81 | 0.92 (0.43-1.96) | 0.84 |
| Body mass index (22≥ kg/m2) | 0.86 (0.41-1.79) | 0.69 | 1.04 (0.48-2.22) | 0.91 |
| Diabetes | 2.13 (0.97-4.71) | 0.05 | 1.99 (0.87-4.55) | 0.1 |
| Hypertension | 1.45 (0.64-3.30) | 0.36 | 1.09 (0.45-2.58) | 0.84 |
| Dyslipidemia | 1.81 (0.87-3.78) | 0.11 | 1.67 (0.78-3.59) | 0.18 |
| History of hospitalization for heart failure | 2.69 (1.22-5.92) | 0.01 | 1.97 (0.82-4.73) | 0.12 |
| Left ventricular ejection fraction (50< %) | 3.87 (1.65-9.07) | 0.001 | 3.09 (1.23-7.77) | 0.01 |

**Supplementary Table 8.**

|  | Univariate | | Multivariable | |
| --- | --- | --- | --- | --- |
|  | HR (95% CI) | P value | HR (95% CI) | P value |
| LPM group | 1.51 (0.42-5.42) | 0.52 | 2.14 (0.43-10.65) | 0.35 |
| Age (80≥ years old) | 2.27 (0.70-7.32) | 0.16 | 6.78 (1.41-32.44) | 0.01 |
| Male | 0.66 (0.23-1.89) | 0.44 | 1.25 (0.32-4.77) | 0.73 |
| Body mass index (22≥ kg/m2) | 0.42 (0.15-1.21) | 0.11 | 0.27 (0.06-1.10) | 0.06 |
| Diabetes | 1.32 (0.41-4.22) | 0.63 | 1.52 (0.39-5.90) | 0.54 |
| Hypertension | 0.27 (0.09-0.81) | 0.01 | 0.12 (0.02-0.59) | 0.008 |
| Dyslipidemia | 0.76 (0.21-2.73) | 0.68 | 1.78 (0.23-13.66) | 0.57 |
| History of hospitalization for heart failure | 1.82 (0.61-5.38) | 0.27 | 4.41 (0.98-19.85) | 0.05 |
| Left ventricular ejection fraction (50< %) | 4.41 (1.39-13.95) | 0.01 | 20.35 (3.57-116.02) | <0.001 |

**Supplementary Table 9.**

|  | **At 0.5 year** | **At 1 year** | **At 2 years** | **At 3 years** |
| --- | --- | --- | --- | --- |
| **LPM group** | 28 (22%) | 19 (15%) | 8 (17%) | 3 (13%) |
| **(N=368)** |  |  |  |  |
| **PM group** | 22 (11%) | 12 (6%) | 9 (7%) | 10 (18%) |
| **(N=561)** |  |  |  |  |

**Supplementary figure legends**

**Supplementary Figure 1.** Kaplan-Meier curves for heart failure hospitalization in patients with sinus node dysfunction (A), atrioventricular block (B), and bradycardic atrial fibrillation (C).

**Supplementary Figure 2.** Causes of worsening heart failure in patients with sinus node dysfunction (A), atrioventricular block (B), and bradycardic atrial fibrillation (C).

**Supplementary Figure 3.** Kaplan-Meier curves for heart failure hospitalization in patients with more or less than 40% cumulative ventricular pacing after pacemaker implantation.

VP, cumulative ventricular pacing.

**Supplementary table legends**

**Supplementary Table 1.** Characteristics in patients ≥82 years

NYHA, New York Heart Association; ACE, angiotensin converting enzyme; ARB, angiotensin II receptor blocker.

**Supplementary Table 2.** Characteristics in patients <82 years

NYHA, New York Heart Association; ACE, angiotensin converting enzyme; ARB, angiotensin II receptor blocker.

**Supplementary Table 3.** Characteristics in patients with sinus node dysfunction for which pacing indication

NYHA, New York Heart Association; ACE, angiotensin converting enzyme; ARB, angiotensin II receptor blocker.

**Supplementary Table 4.** Characteristics in patients with atrioventricular block for which pacing indication

NYHA, New York Heart Association; ACE, angiotensin converting enzyme; ARB, angiotensin II receptor blocker.

**Supplementary Table 5.** Characteristics in patients with bradycardic atrial fibrillation for which pacing indication

NYHA, New York Heart Association; ACE, angiotensin converting enzyme; ARB, angiotensin II receptor blocker.

**Supplementary Table 6.** Univariate and Multivariable Cox Hazard Models for Heart Failure Hospitalization in patients with sinus node dysfunction for which pacing indication

LPM, leadless pacemaker.

**Supplementary Table 7.** Univariate and Multivariable Cox Hazard Models for Heart Failure Hospitalization in patients with atrioventricular block for which pacing indication

LPM, leadless pacemaker.

**Supplementary Table 8.** Univariate and Multivariable Cox Hazard Models for Heart Failure Hospitalization in patients with bradycardic atrial fibrillation for which pacing indication

LPM, leadless pacemaker.

**Supplementary Table 9.** Number of SND patients with more than 40% cumulative ventricular pacing after pacemaker implantation, divided into LPM group and PM group

SND, sinus node dysfunction; LPM, leadless pacemaker; PM, conventional pacemaker.

**Supplementary Figure 1.**

**
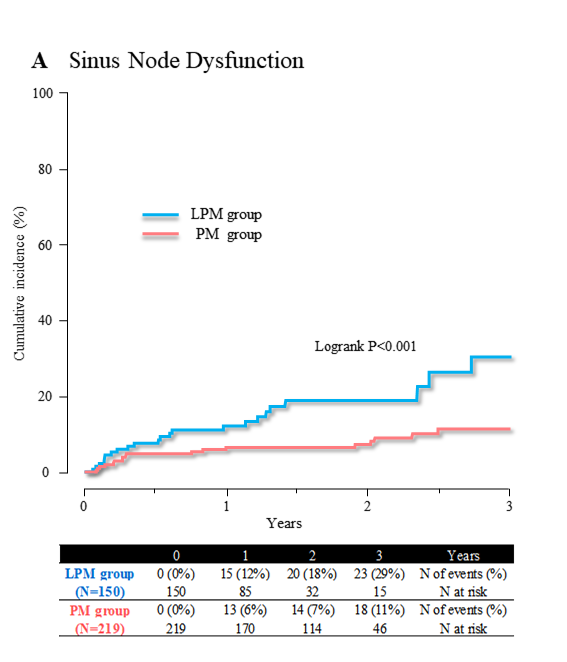
**


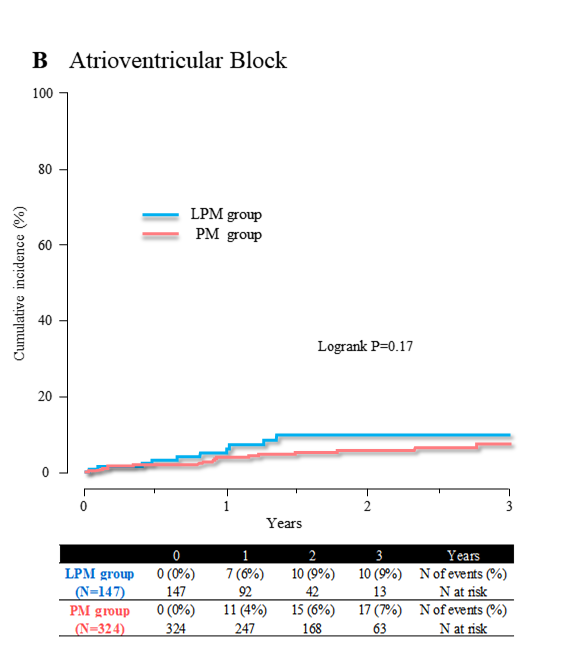


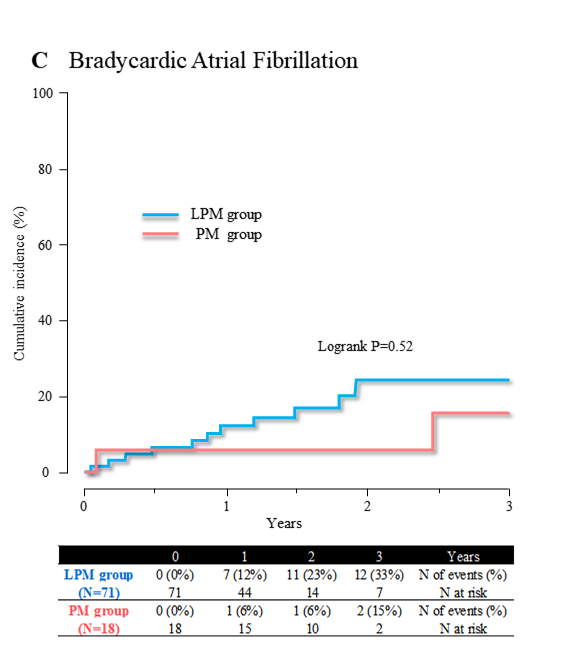


**Supplementary Figure 2.**

**
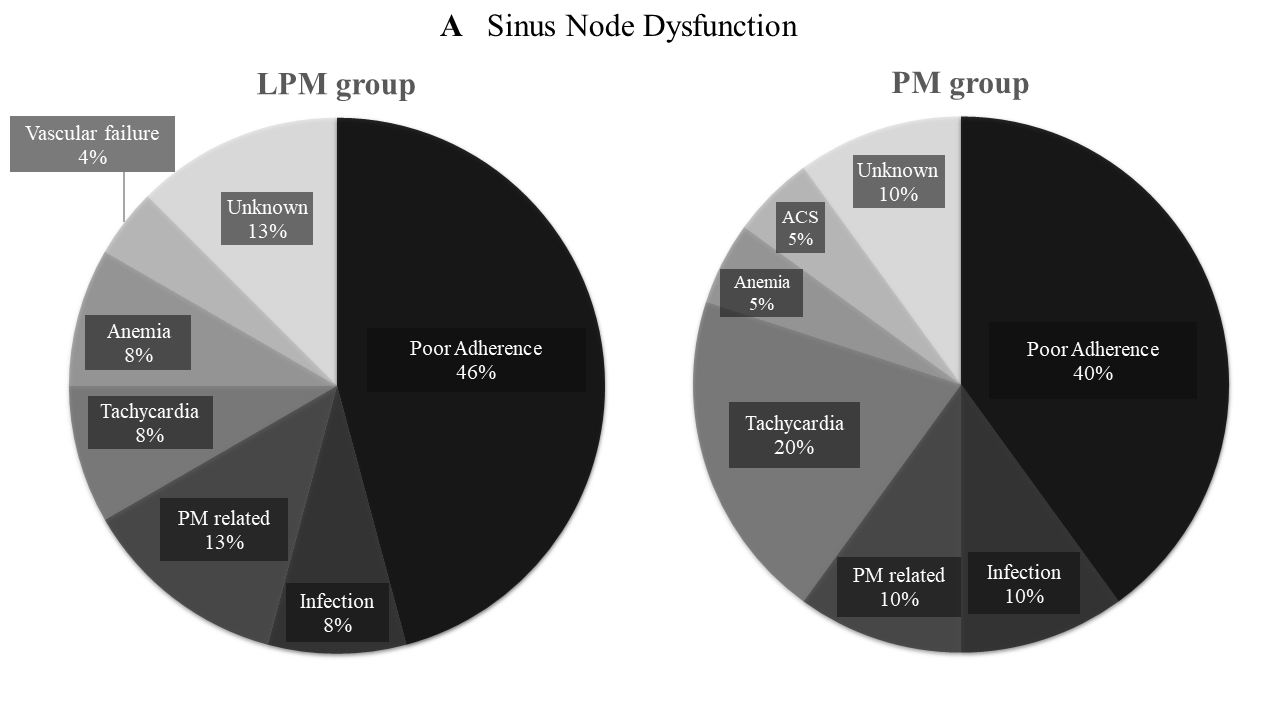
**

**
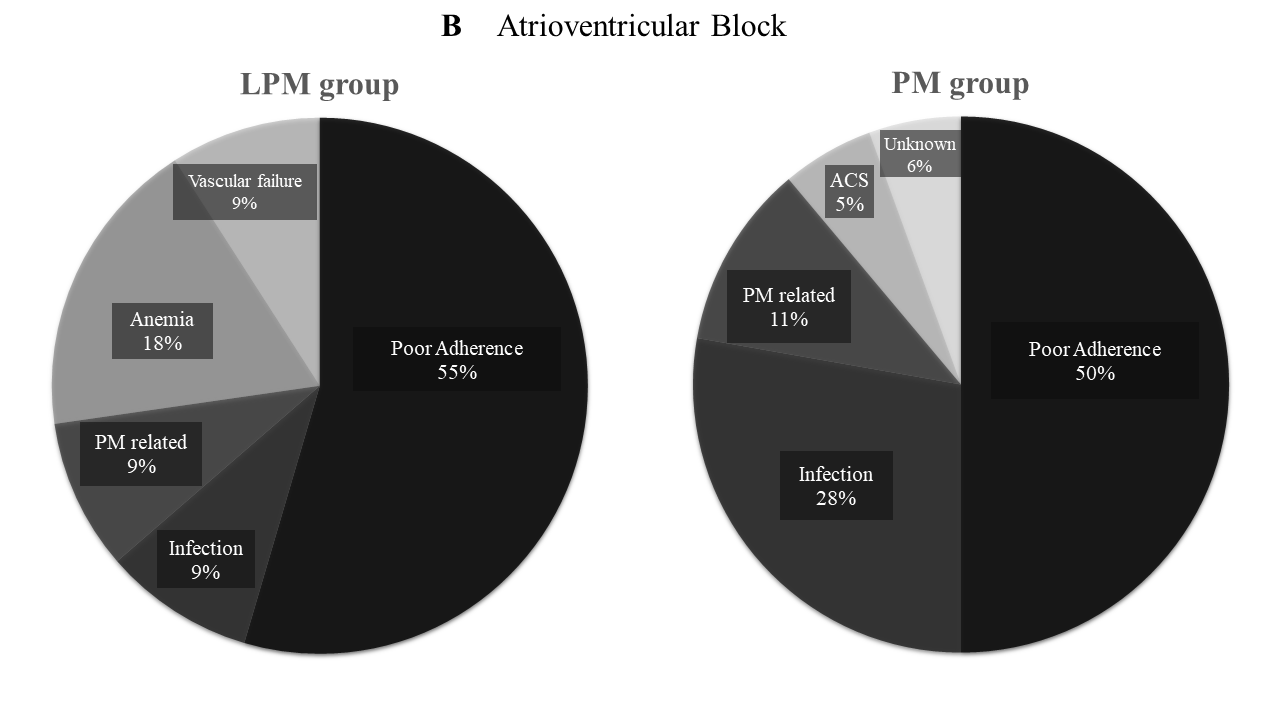
**

**
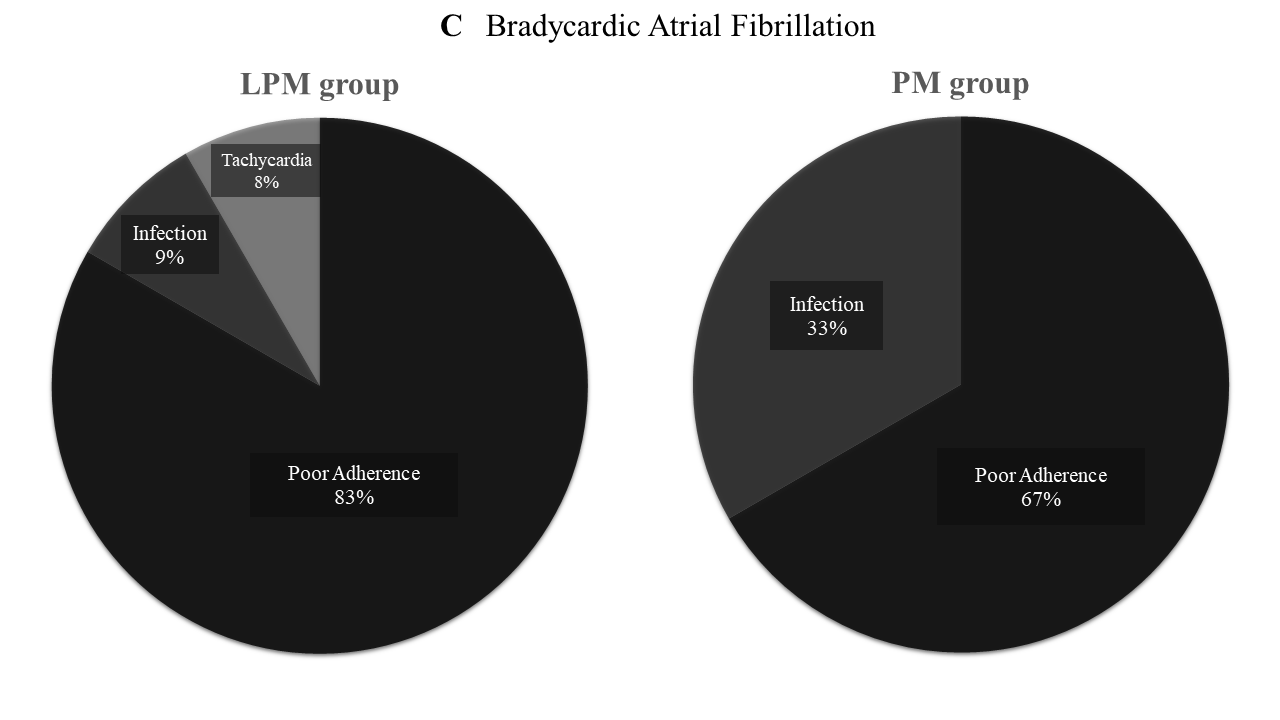
**

**Supplementary Figure 3.**

**
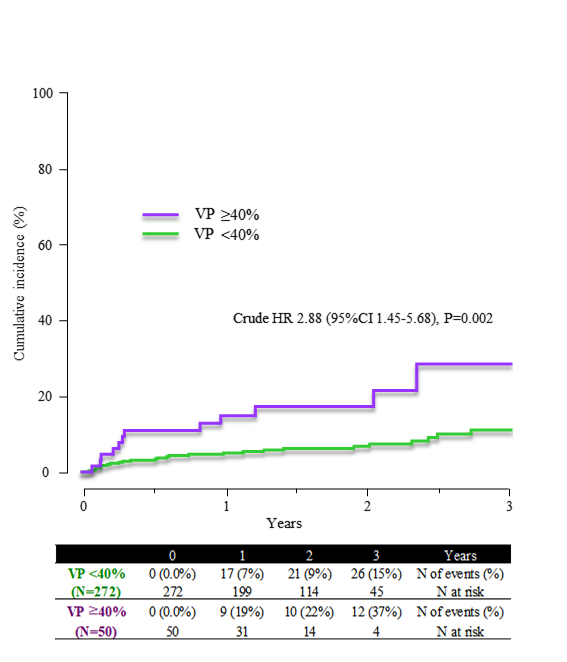
**
